# Can diffusion weighted imaging be used as an alternative to contrast-enhanced imaging on magnetic resonance enterography for the assessment of active inflammation in Crohn disease?

**DOI:** 10.1097/MD.0000000000019202

**Published:** 2020-02-21

**Authors:** Aysegul Cansu, Suleyman Bekircavusoglu, Sukru Oguz, Eser Bulut, Sami Fidan

**Affiliations:** a Karadeniz Technical University, Faculty of Medicine, Department of Radiology; b Trabzon Kanuni Education and Research Hospital, Department of Radiology; c Karadeniz Technical University, Faculty of Medicine, Department of Gastroenterology, Trabzon, Turkey.

**Keywords:** Crohn disease, diffusion weighted imaging, magnetic resonance activity index, magnetic resonance enterography

## Abstract

The present study aimed to investigate the potential use of T2-weighted sequences with diffusion weighted imaging (DWI) in magnetic resonance (MR) enterography instead of conventional contrast-enhanced MR imaging (MRI) sequences for the evaluation of active inflammation in Crohn disease.

Two-hundred thirteen intestinal segments of 43 patients, who underwent colonoscopy within 2 weeks before or after MR enterography were evaluated in this retrospective study. DWI sequences, T2-weighted sequences, and contrast-enhanced T1-weighted sequences were acquired in the MR enterography scan after cleaning of the bowel and using an oral contrast agent. First, the intestinal segments that had active inflammation in MR enterography were qualitatively evaluated in T2-weighted and contrast-enhanced T1-weighted sequences and then MR activity index (MRAI 1) and MRAI 2 were formed with and without contrast-enhanced sequences in 2 separate sessions.

The correlation coefficient between contrast enhanced and DWI MR enterography scores (MRAI 1 and MRAI 2) of intestinal inflammation was 0.97 for all segments. In addition, separate correlation coefficients were calculated for terminal ileum, right colon, transverse colon, left colon, and rectum, and there was a strong correlation between the MRAI 1 and MRAI 2 scores of each segment (*r* = 0.86–0.97, *P* < .001). On the other hand, MR enterography had 88.7% sensitivity, 97.9% specificity, 95.5% positive predictive value, 94.6% negative predictive value, and 94.8% accuracy for detection of active inflammation in all intestinal segments in Crohn disease.

DWI and T2-weighted sequences acquired with cleaning of the bowel can be used instead of contrast-enhanced MRI sequences for the evaluation of active inflammation in Crohn disease.

## 1 Introduction

Crohn disease is a chronic, repetitive inflammatory disease usually observed in the early adulthood period. Diet, smoking, stress, infection, genetic factors, and all autoimmune abnormalities have been considered as etiological factors.^[1]^ Crohn disease predominantly affects the small intestine (up to 80% of the cases) and colon, but any part of the gastrointestinal system can be included in the disease, and more than one region may be affected. The disease is characterized by the formation of erosion, ulceration, full-thickness intestinal wall inflammation, and the formation of granuloma histologically.^[2]^

Close monitoring of disease activity is important because chronic inflammation causes irreversible intestinal damage and complications.^[3]^ Although endoscopy is accepted as the gold standard diagnostic method for the diagnosis and activity evaluation of Crohn disease, it has disadvantages, such as invasiveness, excessive cost, and inadequacy in viewing the small intestine. Evaluation of the intestinal segments between the duodenum and terminal ileum has been problematic, especially in the past, and this was solved with the prevalent use of new methods, such as video capsule endoscopy and enteroscopy.^[4]^ However, the invasive characteristics and excessive costs of these methods do not facilitate wide use in practice. Thus, radiologic methods for the evaluation and follow-up of Crohn disease are of vital importance.

The main radiologic methods used in the evaluation of the small intestine are small intestine–passage radiographies, conventional enteroclysis, ultrasonography, computed tomography (CT) enterography, and magnetic resonance (MR) enterography.^[5]^ Conventional enteroclysis and passage radiographies have been used for many years and are accepted as the main methods for imaging of the small intestine. Information can be gathered about intestinal function and lumen width through these examinations, but a direct examination cannot be performed on the intestinal wall and surrounding tissues. Moreover, their diagnostic accuracy is very limited.^[5]^ CT enterography, which is prevalently used in the diagnosis and follow-up of Crohn disease, provides the opportunity for the evaluation of intestinal and extraintestinal findings;^[6,7]^ however, the content of ionizing radiation causes a problem in the follow-up of young adults and children with a high incidence rate of Crohn disease.

MR enterography is a minimally invasive imaging method that has become increasingly prevalent in recent years, does not involve ionizing radiation, can be easily tolerated by patients, and can show intra-intestinal and extra-intestinal pathologies together.^[8]^ MR enterography has a rate of high accuracy in the diagnosis and determination of activation severity of Crohn disease, and it is particularly important in treatment planning.^[9]^ Today, gadolinium-based intravenous contrast materials are used in conventional MR enterography examinations. These contrast materials have limited safety because they may rarely cause allergic reactions and nephrogenic systemic fibrosis in kidney function disorders, although they have a much lower allergy risk compared with iodized contrast materials, and they are not nephrotoxic.^[10]^ The existence of a higher rate of renal function disorders in Crohn disease compared with the normal population as well as the fact that most patients are young increases the long-term exposure risk of this group to gadolinium.^[11]^ Moreover, in recent years, there have been studies on the accumulation of linear gadolinium-based contrast agents in the neuronal tissues with repeat use.^[12]^ The cost of the examination also increases with the use of contrast agents. All of these disadvantages of contrast agents have resulted in alternative no-contrast MR enterography examinations for patients with Crohn disease who need to be monitored with MR enterography many times throughout their lives. Diffusion-weighted imaging (DWI) is being used as a promising MR method for the evaluation of active inflammation in Crohn disease.^[13–16]^ DWI is an MR examination method in which the diffusion of extracellular fluid is measured quantitatively and stated as the apparent diffusion coefficient (ADC) value. In cases such as malignity and inflammation, in which cellularity increases, a restriction occurs in the diffusion of extracellular fluid. It is known that the active intestinal inflammation observed in Crohn disease causes the restriction observed in DWI (ie, the decrease in ADC values), although the definitive microscopic basis for diffusion restriction is not adequately understood.^[17]^ To our knowledge, there are very few studies in the literature comparing DWI and conventional contrast-enhanced magnetic resonance imaging (MRI) sequences and studying the diagnostic effectiveness of DWI in the evaluation of active inflammation in Crohn disease.^[13,18]^

The present study aimed to investigate the potential use of non-contrast T2-weighted sequences with DWI in MR enterography instead of conventional contrast-enhanced MRI sequences for detecting active inflammation in Crohn disease.

## 2 Materials and methods

### 2.1 Study population

In total, 48 adult patients who were admitted to our university hospital with active complaints between 1 October 2015, and 31 October 2017, were diagnosed with Crohn disease via colonoscopy and histopathology, and underwent colonoscopy within 2 weeks before or after MR enterography were included in our study. Of these patients, 2 patients without diagnostic colonoscopy results due to inadequate cleaning of the bowel cleaning, 2 patients with inadequate image quality, and 1 patient with a history of contrast agent allergy and without contrast-enhanced MR enterography were excluded from the study. Patient informed consent was waived in this retrospective study, which was approved by the local ethical committee.

### 2.2 MR enterography protocol

MR enterography scans were performed for 43 patients with a 3-Tesla (3T) MR imaging (MRI) system (Magnetom, Skyra; Siemens Healthcare, Erlangen, Germany). The patients were recommended to consume juicy foods 1 day prior to the scan and were given 300 mL sennoside A + B calcium solution (XM; solution 150 mL, Yenisehir Lab, Ankara, Turkey) for cleaning of the bowel 12 hours prior to the scan. Our patients were asked to fast for 6 to 8 hours prior to MR examination. The patients were given 1500 mL 3% mannitol solution (500 mL every 15 minutes) for intestinal distention 45 minutes before the scan. Further, 20 mg hyoscine N-butylbromide (Buscopan, Boehringer Ingelheim, Eczacibasi, Turkey) was administered intravenously before MR enterography to decrease intestinal peristalsis. MRI examination was initiated after adequate distention was observed in the pilot images.

For the upper and lower abdomen, axial T2-weighted half-Fourier single-shot fast spin-echo sequence (HASTE: repetition time [TR], 1400 ms; echo time [TE], 95 ms; field of view [FOV], 380 mm; slice thickness, 6 mm; slice gap, 1.2 mm; matrix, 203 × 320), coronal T2-weighted HASTE (TR, 1200 ms; TE, 91 ms; FOV, 400 mm; slice thickness, 5 mm; slice gap, 1 mm; matrix, 256 × 320), axial fat suppressed T2-weighted HASTE ( TR, 1400 ms; TE, 95 ms; FOV, 380 mm; slice thickness, 6 mm; slice gap, 1.2 mm; matrix, 203 × 320), coronal T2-weighted steady-state gradient echo sequence (TRUFI: TR, 570 ms; TE, 1.68 ms; FOV, 380 mm; slice thickness, 5 mm; slice gap, 0.5 mm; Flip Angle, 55°; matrix, 256 × 256), axial DWI (with b factors of 50, 400, and 800 s/mm^2^; TR, 4200 ms; TE, 53 ms; FOV, 380 mm; slice thickness, 6 mm; slice gap, 1.2 mm; matrix, 108 × 134) were acquired. Further, 0.2 mL/kg gadoterate meglumine (Dotarem; Guerbet, Villepinte, France) was intravenously administered via an automatic injector at a rate of 2 mL/s, followed by a 20-mL saline flush. Images were acquired including T1-weighted 3-dimensional (3D) gradient echo sequences with fat suppression in the arterial, portal venous, and late venous phase on the coronal plane and in the late venous phase on the axial plane T1-weighted 3D gradient echo (coronal T1 VIBE: TR, 4.21 ms; TE, 1.34 ms; FOV, 450 mm; slice thickness, 1.5 mm; slice gap, 0.3 mm; matrix, 208 × 320; and axial T1 VIBE: TR, 3.97 ms; TE, 1.29 ms; FOV, 380 mm; slice thickness, 3 mm; slice gap, 0.6 mm; matrix, 195 × 320;). Finally, the DWI sequence was acquired in the coronal plane with b factors of 50, 400, and 800 s/mm^2^ (TR, 4900 ms; TE, 58 ms; FOV, 400 mm; slice thickness, 6 mm; slice gap, 1.2 mm; matrix, 108 × 134). The average examination time was 40 minutes.

### 2.3 Ileocolonoscopy

Colonoscopy was performed using video colonoscopy (CF H260AL; Olympus, Japan) by 2 experienced gastroenterologists after the patients were given 300 mL sennoside A + B calcium solution (XM; solution 150 mL, Yenisehir Lab, Ankara, Turkey) for cleaning of the bowel 12 hours prior to the application.

### 2.4 MR enterography analysis

In MR enterography examinations, the intestines were separated into 5 segments (terminal ileum, right colon, transverse colon, left colon, and rectum), and 213 inflamed or noninflamed intestinal segments of 43 patients were evaluated (hemicolectomy existed in 2 patients). The evaluation was performed in two separate sessions by the agreement of two radiologists who were not informed about the colonoscopy results. An interval of 1 month between the 2 sessions was designed to avoid recall bias. In the first session, intestinal segments that had active inflammation and were normal in MR enterography were qualitatively evaluated in T2-weighted and contrast-enhanced T1-weighted sequences. Moreover, in the first session, the following parameters were assessed according to the methods formed in previous studies:^[8,13,18]^ intestinal wall thickness, mural T2 signal (compared with the psoas muscle), perimural T2 signal, comb sign (in contrast-enhanced examinations), and mural contrast enhancement degree in the venous phase, and a score was assigned for each parameter. In the second session conducted 1 month later, intestinal wall thickness, mural T2 signal, perimural T2 signal, and comb sign (in the TRUFI) were evaluated. Scoring was performed according to the diffusion restriction in the DWI sequences instead of the contrast-enhanced sequences (Table 1). MR activity indexes (MRAI 1 and MRAI 2) were formed by adding the points given in both sessions. Moreover, the existence, number, and short axis measurement of lymph nodes; the length of the involved segment; the mesenteric fibrofatty proliferation; and the existence of fistula and stricture were evaluated.

**Table 1:**
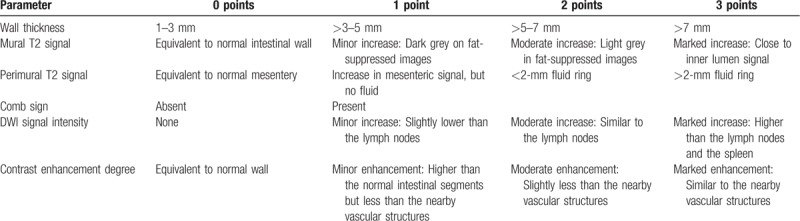
Magnetic resonance activity index scores.

### 2.5 Statistical analysis

The Statistical Package for the Social Sciences (SPSS) 23.0 was used for data analysis (IBM, New York, NY). Descriptive statistics of the evaluation results were given as numbers and percentiles for categorical variables and mean value, standard deviation, minimum value, and maximum value for measured variables. One-sample Kolmogorov-Smirnov test was used to analyse the normal distribution of the data. Because the measured variables did not have a normal distribution, correlation coefficients and statistical significance were calculated using Spearman rank correlation analysis. The measured data of 2 dependent groups were compared using the Wilcoxon test. McNemar test was used in the analysis of the differences between the rates of categorical values in the dependent groups. Kappa test was used in the evaluation of reliability within the dependent groups. The sensitivity, specificity, positive predictive and negative predictive values, and accuracy of MR enterography in diagnosing Crohn disease were calculated. The level of statistical significance was accepted as *P* < .05.

## 3 Results

There were 43 patients in our study, of whom 28 were male (65.1%) and 15 were female (34.9%), and their ages varied between 18 and 72 (mean, 34.3 ± 13) years. All patients had colonoscopic and histopathological diagnosis of Crohn disease. In total, 142 segments were normal and 71 segments were compatible with active inflammation in the colonoscopy. Furthermore, 147 segments were evaluated as normal, and 66 segments were evaluated in favour of active inflammation on MR enterography examination. In 8 segments, colonoscopic findings of active inflammation existed, whereas these segments were evaluated as normal on MR enterography. In 3 segments, there were normal findings on colonoscopy, whereas these segments showed active inflammation findings on MR enterography (Table 2).

**Table 2:**
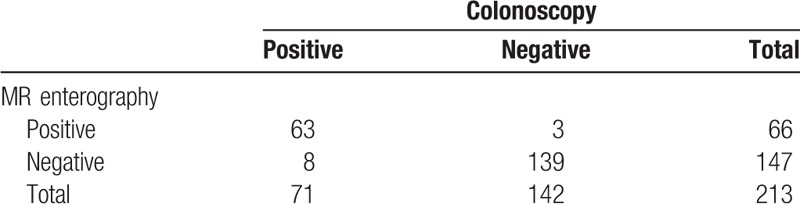
Crohn disease findings in colonoscopy and magnetic resonance enterography.

In our study, MR enterography for the detection of active inflammation in Crohn disease had, according to all segments, 88.7% sensitivity, 97.9% specificity, 95.5% positive predictive value, 94.6% negative predictive value, and 94.8% accuracy. There were 43 terminal ileum, 43 right colon, 43 transverse colon, 42 left colon, and 43 rectum involvements existing in the segmental evaluation. Diagnostic values of MR enterography according to the segments are presented in Table 3.

**Table 3:**
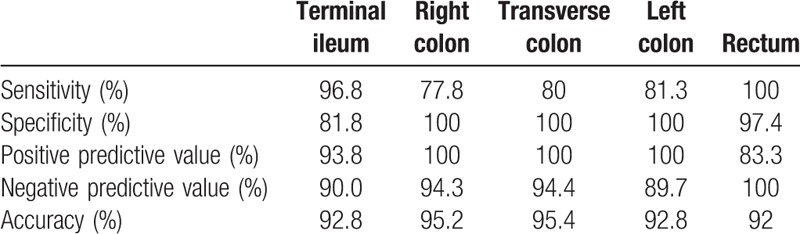
Diagnostic validity of magnetic resonance enterography according to segments in Crohn's disease.

**Figure 1:**
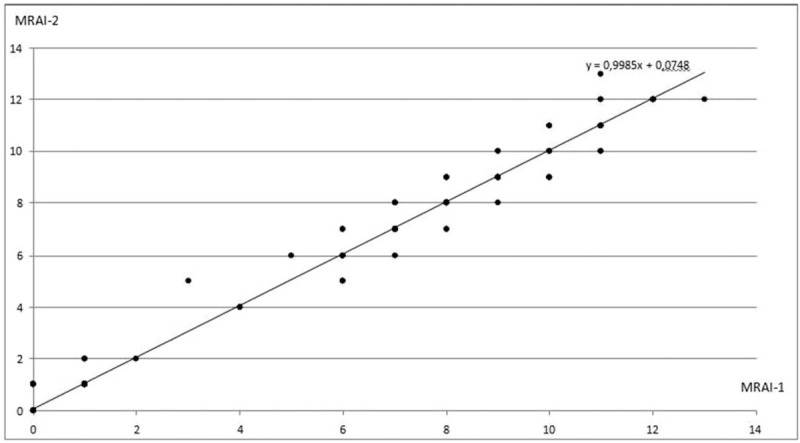
Graph showing the correlation between MRAI 1 and MRAI 2. MRAI = magnetic resonance activity index.

**Figure 2:**
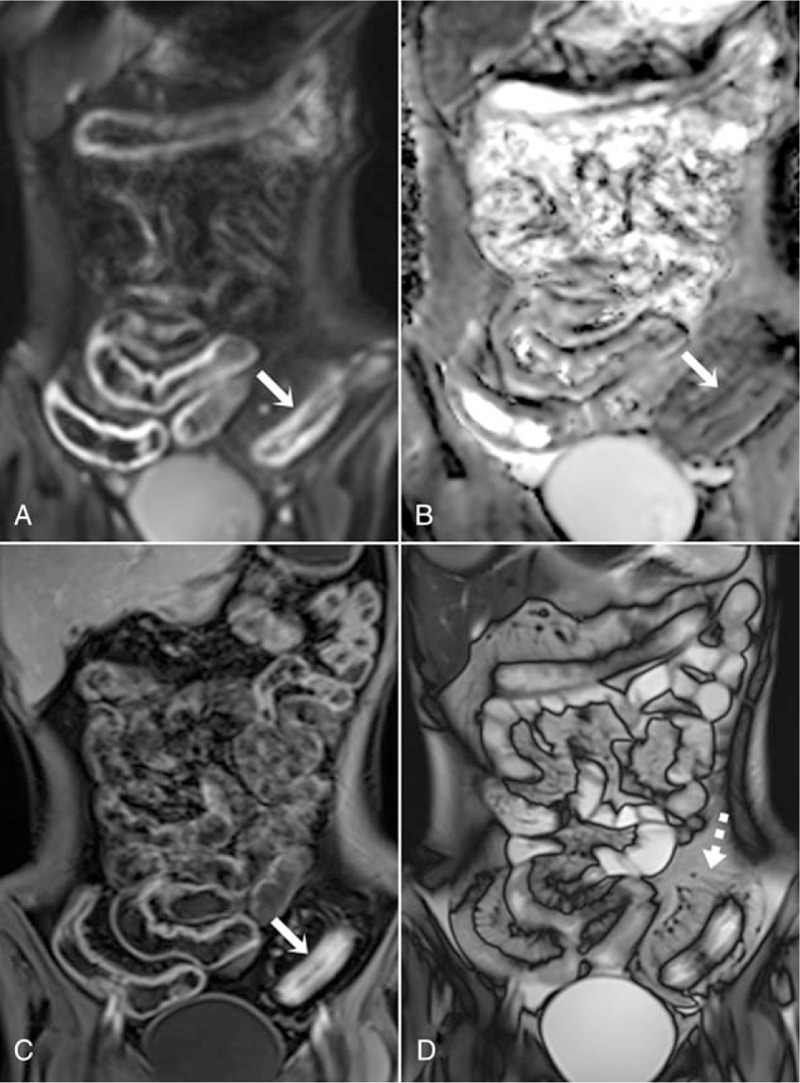
MR enterography images of an 18-yr-old female patient with severe active Crohn disease. Diffusion restriction is observed in the sigmoid colon in the coronal DWI (A) and ADC map (B**)** (arrows). Because the DWI signal increase is higher than in the lymph nodes, it scored 3 points on the MRAI. On the contrast-enhanced coronal T1-weighted image (C) of the same patient, mural thickening of the sigmoid colon and hyperenhancement that is more apparent in mucosa are seen (arrow). Because contrast enhancement is similar to that of the nearby vascular structures, it was scored as 3 points. Moreover, comb sign is observed on the coronal TRUFI sequence (D) (dashed arrow). ADC = apparent diffusion coefficient, DWI = diffusion weighted imaging, MR = magnetic resonance.

**Figure 3:**
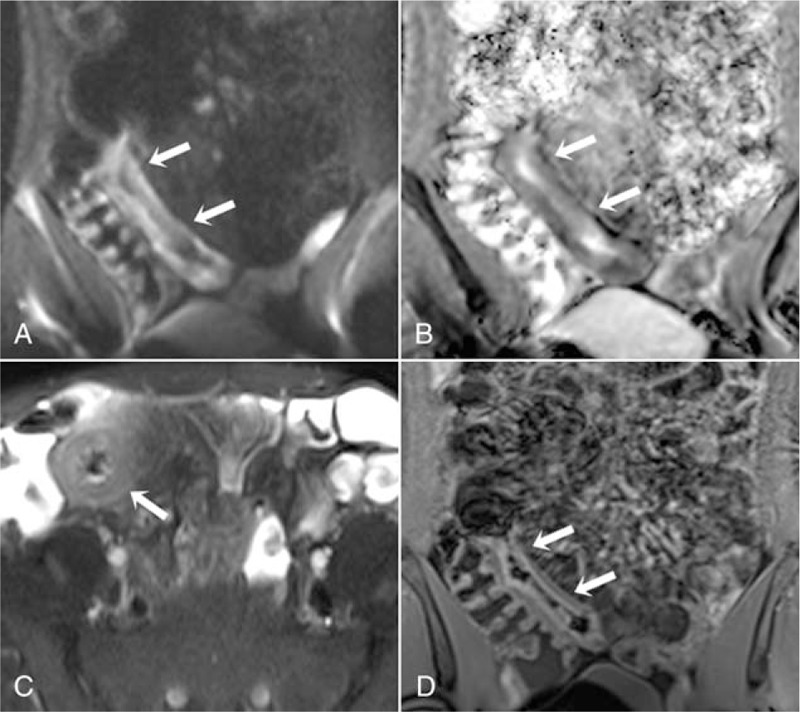
A 30-yr-old male patient with active Crohn disease. Diffusion restriction is observed in the distal and terminal ileum in the coronal DWI (A) and ADC map (B) (arrows) (scored 2 points on the MRAI). On the axial T2-weighted images (C) of the same patient, wall thickness, signal increase secondary to oedema, and perimural fluid ring are observed in the terminal ileum (arrow). On the coronal contrast-enhanced T1-weighted images (D), mucosal hyperenhancement is seen (scored 2 points on the MRAI). ADC = apparent diffusion coefficient, DWI = diffusion weighted imaging, MR = magnetic resonance.

**Figure 4:**
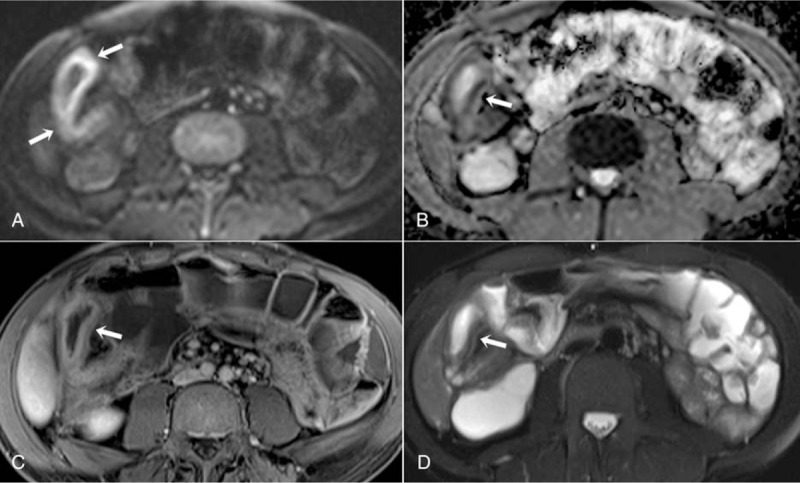
MR enterography findings of a 37-yr-old male patient show active inflammation in the right colon (arrows). Marked diffusion restriction is observed in the intestinal wall in the right colon on axial DWI (A) and ADC map (B) (scored 3 points). Mucosal hyperenhancement is observed on contrast-enhanced axial T1-weighted image (C), and wall thickness is observed on axial fat-suppressed T2-weighted image (D**)**. Because contrast enhancement was less than the nearby vascular structure, it was scored as 2 points. ADC = apparent diffusion coefficient, DWI = diffusion weighted imaging, MR = magnetic resonance.

In the MRAI in both sessions, the score changed in the range of 0 to13 (mean, 3.06) in the first session and 0 to 13 (mean, 3.99) in the second session. The correlation coefficient of the intestinal inflammation severity between contrast and DWI MR enterography scores (MRAI 1 and MRAI 2) was 0.97 (*P* < .001) for all segments, indicating a strong positive correlation. The contents of these scores and correlation graph are shown in Figures 1–4. In addition, separate correlation coefficients were calculated for terminal ileum, right colon, transverse colon, left colon, and rectum, and there was a strong correlation detected between the MRAI 1 and MRAI 2 scores of each segment (*r* = 0.86–0.97, *P* < .001). Correlation coefficients and *P* values are presented in Table 4.

**Table 4:**
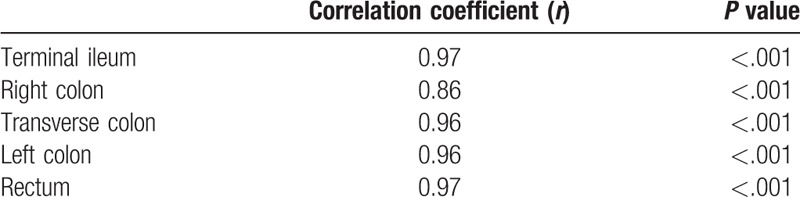
Magnetic resonance activity index 1 and magnetic resonance activity index 2 correlation according to segments.

Lengths of the segments showing active Crohn involvement changed in the range of 1 to 26 cm (mean, 8.6 cm). Wall thickness in the segments showing involvement was found to be 3.4 to 15 mm (mean, 7.58 mm) in the first session and 3.6 to 14 mm (mean, 7.57 mm) in the second session. There was no statistically significant difference detected in the wall thickness measurements between the first and second sessions.

**Figure 5:**
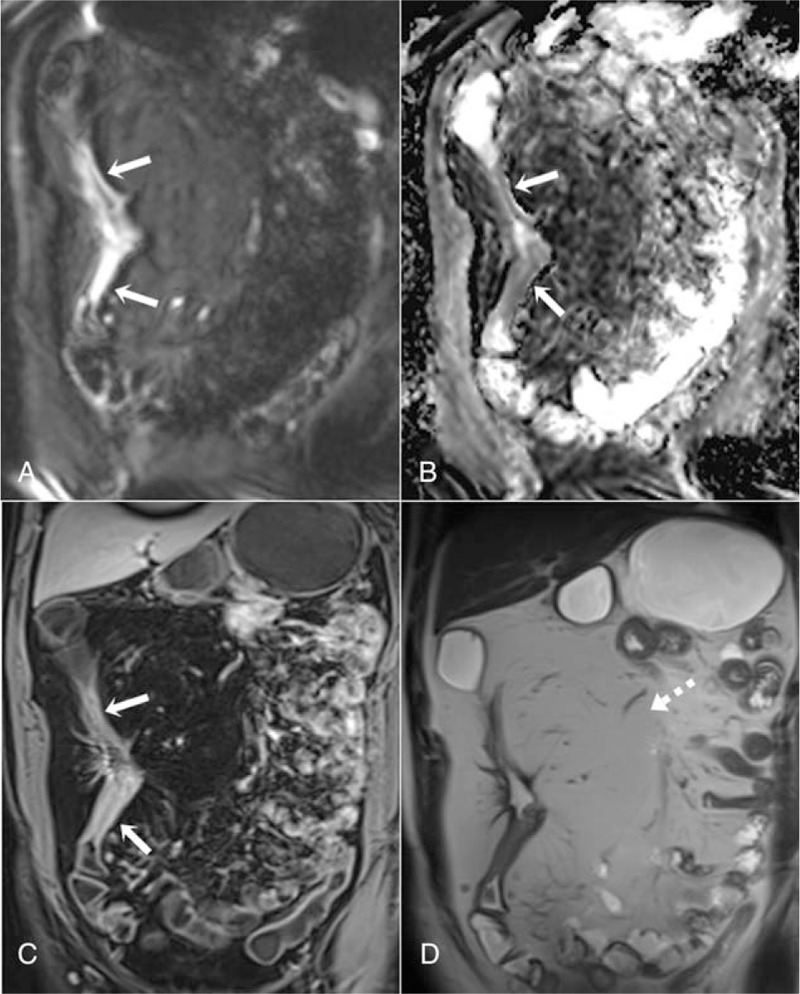
A 30-yr-old male patient with active Crohn disease on a chronic basis in the descending colon. Diffusion restriction is observed in the colon wall on coronal DWI (A) and the ADC map (B**)** (arrows). Wall thickness, increased contrast enhancement, and strictures are observed on the coronal contrast-enhanced T1-weighted sequence (C**)** (arrows). Fibrofatty proliferation exists neighbouring the pathological intestinal segment on the coronal T2-weighted images (D**)** (dashed arrow). ADC = apparent diffusion coefficient, DWI = diffusion weighted imaging.

More than 1 segmental involvement was observed in 17 patients. Fibrofatty proliferation was detected in a total number of 54 segments. Fistula was detected in 20 patients (perianal in 15 patients and enteroenteric in 5 patients). Stricture was observed in 12 segments (Fig. 5).

When the DWIs were evaluated in terms of fistula diagnosis, 13 of 20 patients with fistula were diagnosed with fistula (65%). However, fistula location was not precisely determined in most of them.

Multiple mesenteric lymph nodes existed in 21 patients, fewer than 3 mesenteric lymph nodes existed in 17 patients, and mesenteric lymph nodes were not observed in 5 patients. Short axes of mesenteric lymph nodes were found to be in the range of 3 to 17 mm (mean, 7.23 mm).

Mural T2 hyperintensity and perimural T2 hyperintensity were evaluated in both sessions, and a strong reliability existed in both sessions (kappa values = 0.99 and 0.98, respectively).

Comb sign was evaluated in both sessions and was detected in 58 segments in the first session and 56 segments in the second session. There was a strong intra-observer reliability in comb sign detection (kappa value = 0.84, *P* < .001).

## 4 Discussion

We found a strong correlation between the MRAIs obtained in separate sessions performed to determine the severity of inflammation in our study, which compared the diagnostic effectiveness of standard T2-weighted MR sequences with DWI and contrast-enhanced MRI sequences in the evaluation of active inflammation via MR enterography for patients with a diagnosis of Crohn disease. Moreover, the diagnostic accuracy of MR enterography in general and according to segments was found to be quite high in our study, in which colonoscopy was used as the reference method.

Standard MR enterography protocols in the evaluation of active inflammation in Crohn disease include T2-weighted and fat-suppressed contrast-enhanced T1-weighted sequences.^[17–20]^ After the distention of the intestines is achieved using water or other biphasic inner lumen contrast materials, the contrast enhancement difference between the intestinal wall increases with the administration of intravenous contrast agent, and the pathology and contrast enhancement pattern in the intestinal wall is more clearly evaluated.^[21]^ Intestinal wall contrast enhancement in inflammatory bowel diseases during the transition from the active phase to the remission phase shows similarities to a normal intestinal wall. Intestinal wall thickening was also present in both phases. However, wall thickness was observed in the active phase due to oedema and inflammation, and it persists during the chronic phase due to fibrosis.^[22]^ DWI is used in clinical practice mostly as an addition to standard MR enterography sequences, as it takes a shorter time and does not require contrast agents. However, there are contradicting results in the literature regarding its diagnostic effectiveness, contribution to conventional contrast MR enterography, and the alternative use of DWI MR enterography.^[13,18,23–27]^ In a study by Seo et al, in which the authors evaluated 171 small intestinal segments in 44 patients with Crohn disease, no significant difference was found between contrast-enhanced MR enterography and DWI MR enterography examinations in terms of sensitivity and specificity in detecting the inflammation in the terminal ileum.^[18]^ The MR enterography scores were calculated in that study in a manner that was similar to ours, that is, by the measurement of the intestinal wall thickness during active inflammation, mural T2 signal, perimural T2 signal, and the degree of diffusion restriction and contrast enhancement strength in different sessions. These scores were compared with the Crohn disease endoscopic index of severity (CDEIS)^[28]^ in this study, and a high correlation was found between CDEIS and both DWI MR enterography index of severity and contrast-enhanced MR enterography index of severity.^[18]^ In our study, all segments of 43 patients that could be evaluated through colonoscopy were examined with MR enterography. Furthermore, 71 pathological segments and 142 normal segments according to colonoscopy were included in the study. The evaluation was performed in two different sessions, with a 1-month interval in between. There was a strong correlation between the activity indices formed in these sessions (correlation coefficient, 0.97). Diffusion restriction was detected in all segments that were pathologic on colonoscopy. However, a comparison was not performed between MR enterography activity indices and the endoscopic severity indices in our retrospective study. Conversely, colon segments including terminal ileum were evaluated in our study, and the diagnostic efficiency of DWI MR enterography in colon segments was also analysed.

Because there is an involvement of the colon often along with the small intestine in Crohn disease, radiologic examination of the colon is of high importance. In our study, intestinal segments that could be evaluated by colonoscopy were separated into 5 different segments as the terminal ileum, right colon, transverse colon, left colon, and rectum, and similar correlation coefficients were detected in contrast-enhanced MR enterography and DWI MR enterography. However, in most of the studies in the literature, the most frequent false-positive DWI results were in the colorectal region.^[13,14,27]^ In these studies, which were performed by giving an oral contrast agent only after a period of hunger, bowel cleaning and liquid diet were not applied. The hyperintensity of high-density intestinal content can continue even in high b values in DWI and can cause a false-positive diagnosis.^[15]^ To our knowledge, very few studies in the literature have performed bowel cleaning in MR enterography.^[15,29–31]^ We believe that this also has an effect in contradicting the results regarding diffusion-weighted MR enterography. Moreover, inadequate intestinal distention also causes false-positive DWI results.^[13]^ In our study, which used bowel cleaning and an MR enterography examination toward the upper and lower abdomen that averaged 40 minutes long, we believe that acquiring DWI sequences taken in the axial plane at the beginning of the examination and acquiring the DWI sequence taken in the coronal plane at the end of the examination contributed to ensuring adequate distention in the colon segments and a strong correlation with the contrast-enhanced examinations. Despite the argument that the colon distention problem can be solved by administering a rectal contrast agent that is not comfortable for the patient^[31,32]^ in order to decrease false-positive DWI, we believe that taking the DWI sequence at the beginning of the examination will decrease false positivity in the jejunal intestinal segments, and taking the DWI sequence at the end of the examination will decrease the false positivity in the colon.

In our study, diffusion restriction in DWI MR enterography was qualitatively evaluated, and MR activation scoring was performed using a method similar to previous studies for the purpose of standardization by comparing with lymph nodes and spleen.^[13]^ Conversely, there are many studies indicating a correlation between the Crohn disease activity index and ADC values and providing quantitative evaluation.^[18,31,32]^ However, thick DWI slices and intestinal peristalsis, especially when the intestinal wall is not very thick, frequently make ADC measurement difficult. In a study by Pendse et al, weak intra-observer reliability in the ADC values supports the proposal that the use of this method in practice is not very possible.^[33]^

One of the most important results of the activation in Crohn disease is the contrast enhancement of intestinal walls. In our study, wall contrast enhancement scoring was performed by comparing the nearby vascular structures used in the previous studies.^[8,34]^ This method used in MR enterography is qualitative, and acquiring significant results in the quantitative evaluation of CT enterography shows that the degree of contrast enhancement plays an important role in determining activation.^[35]^ In a study by Qi et al, 88 Crohn disease patients were separated into two groups: active and in remission. Wall thickness and degree of contrast enhancement were compared with CT enterography and statistically significant difference was found between groups.^[35]^ In our study, there was a statistically significant difference when the mural contrast enhancements of normal and inflamed intestines were compared (*P* < .001). There were 3 segments with minor enhancement, 2 segments with moderate enhancement, and 1 segment with marked enhancement on MR enterography with negative colonoscopy findings. The existence of nonspecific colitis findings on colonoscopy and histopathology in 3 segments and inadequate intestinal distention in the other 3 segments can be counted among the reasons.

Inflammatory findings accompany the peripheral mesentery along with intestinal wall inflammation in Crohn disease. Mesenteric lymphadenopathies, mesenteric fibrofatty proliferation, and comb sign indicating an increase in vascularity show that there is transmural inflammation.^[36]^ Meyers et al defined the increase of mesenteric vascularity as comb sign and indicated it as an important result in terms of the activity of Crohn disease.^[37]^ However, this sign is not pathognomonic and can also be observed in other forms of acute enterocolitis.^[37]^ In our study, comb sign was detected in 75% and mesenteric fibrofatty proliferation was detected in 69% of the segments that were found to be compatible with active Crohn disease via colonoscopy. In the second session when the DWI MR enterography was analysed, comb sign was evaluated in TRUFI, and diagnostically, there was no significant difference found when compared with the contrast-enhanced sequences. Thus, we believe that there is no need for contrast material to evaluate this finding.

In our study, another factor contributing to the high diagnostic accuracy of MR enterography was that a 3T MRI scan was used. There are very few studies on MR enterography conducted with a 3T MRI scan, and in these studies, MR enterography was found to have high accuracy in the determination of active inflammation.^[13,16,18,31]^ Images acquired with the 3T MRI scan have better signal and spatial resolution compared with the images acquired with 1.5T. This contributes to the increase in sensitivity in the detection of lesions.^[38]^ Conversely, it is known that connected to the effect of the increased magnetic field force in 3T MRI machines, the susceptibility artifact becomes more apparent in T2-weighted sequences. This may be perceived as a disadvantage, but because the inflammatory changes in the intestinal wall are usually observed in the longer segment, we believe that these artifacts do not cause a limitation in the detection of inflammatory changes. Moreover, as in our study, fast turbo spin-echo sequences can be used to decrease the susceptibility artefact.^[39]^

The results of our study bring forth the idea that an MR enterography examination acquired without the administration of intravenous contrast material with the use of DWI is an applicable option to evaluate active inflammation in intestinal segments of patients with Crohn disease. In this study, DWI and contrast-enhanced MR examinations provided similar results in the detection of active inflammation, but the detection of all findings observed in Crohn disease could not be performed through only DWI. Enteroenteric fistula was observed in 5 patients and perianal fistula was observed in 15 patients on the contrast-enhanced MR examination. Only 13 (65%) of the detected 20 fistulas were able to be detected with DWI. The locations of the perianal fistulas that could be detected with DWI were unable to be precisely determined. It is accepted that contrast-enhanced MR enterography is a reliable diagnostic tool in the diagnosis of penetrating complications.^[40]^ Larger studies are needed on the use of DWI in the diagnosis of penetrating complications.

The present study has some limitations, including its retrospective nature and lack of surgical, clinical and laboratory findings. The sample size was inadequate and there was no control group in our study showing a lack of intestinal disease. Because the small intestinal segments beyond terminal ileum could not be evaluated by colonoscopy, they were not included in this study. However, high diagnostic accuracy of our results suggest DWI can be used for the evaluation of small bowels on MR enterography. We believe that it is necessary to compare DWI MR enterography and enteroscopy or video capsule endoscopy with further prospective studies. On the other hand, we did not evaluate intestinal fibrosis and stricture in this study. Unrestricted diffusion is frequently seen in fibrosis,^[41]^ however a few studies have reported that mural fibrosis may show diffusion restriction.^[42,43]^ Therefore future studies are needed on the use of DWI in the diagnosis of fibrosis. Finally, the correlation between the MRAIs we formed and CDEISs could not be calculated because of the retrospective nature of our study.

## 5 Conclusions

In conclusion, DWI and T2-weighted sequences performed along with bowel cleaning and acquired at different times can be used instead of contrast-enhanced MRI sequences in the detection of active inflammation in Crohn disease.

## Author contributions

**Data curation:** Suleyman Bekircavusoglu, Eser Bulut.

**Investigation:** Aysegul Cansu, Suleyman Bekircavusoglu.

**Methodology:** Sukru Oguz.

**Resources:** Sami Fidan.

**Writing – original draft:** Aysegul Cansu.
